# Prioritizing challenges in AI adoption for the legal domain: *A systematic review and expert-driven AHP analysis*

**DOI:** 10.1371/journal.pone.0326028

**Published:** 2025-06-24

**Authors:** Sihyun Kim, Sangyoon Yi, Sung-Pil Park

**Affiliations:** Graduate School of Future Strategy, KAIST, Daejeon, Republic of Korea; Hamad Bin Khalifa University College of Science and Engineering, QATAR

## Abstract

This research explores the crucial challenges influencing the adoption of Artificial Intelligence (AI) in the legal domain, a field facing escalating challenges due to rapid technological advancements. We have comprehensively identified, extracted, and evaluated 11 pivotal factors across legal, technical, and socio-ethical dimensions through a systematic review based on the PRISMA guideline. These factors are categorized into three principal groups. Utilizing an analytic hierarchy process (AHP), our innovative approach assesses the relative importance of these challenges based on data meticulously gathered from eight domain experts in law and AI. Our findings pinpoint legal aspects as the paramount category, with liability as the foremost concern among the analyzed factors. These insights offer robust and actionable guidelines for integrating AI into legal practices and underscore this study’s unique contribution to bridging the gap between legal professionals and technology developers. By highlighting the practical applications of our results, this paper facilitates a deeper understanding and proactive engagement with the essential considerations pivotal for the future adoption and evolution of AI within the legal domain.

## 1. Introduction

The rapid advancement of artificial intelligence (AI) in recent years has garnered significant attention because of its profound impact on society and the economy. As AI penetrates into various sectors, both high hopes and concerns emerge regarding its extensive societal repercussions. Particularly in the legal realm, the integration of AI is anticipated to catalyze groundbreaking advancements [1], with the potential to permeate nearly every aspect of legal practices [2]. Beyond enhancing legal services, AI’s application extends into public sectors, serving as a crucial aid in legal decision-making processes [3]. AI technologies have notably advanced to the point where systems have been developed to support human judges in reaching legal judgments [4]. The necessity for innovation and adaptation is increasingly pressing within the legal sector [5], as AI’s influence extends from legal services to potentially enhancing the efficiency of judicial systems [6].

For the purposes of this paper, AI refers specifically to modern computational techniques such as machine learning, natural language processing, and predictive analytics, which have evolved considerably in recent decades. While earlier AI systems, such as rule-based expert systems, laid the groundwork for these technologies, our focus is on advancements made after the year 2000, reflecting the current landscape of AI applications in the legal sector.

Traditionally, the legal industry has shown reluctance to adopt technological innovations at a pace comparable to other fields of knowledge [7]. The introduction of AI is expected to bring substantial changes to conventional legal operations [8]; however, this swift integration of AI is expected to open avenues for merging and creating new services that add value [9]. As the legal sphere prepares for these novel changes, it encounters the challenge and opportunity of utilizing AI to significantly improve the legal profession and the judicial system.

For AI systems to be effective within the legal domain, careful consideration of the characteristics of the applied domain is essential [10]. This involves a meticulous evaluation of the suitability and appropriateness of AI adoption within the legal sector and an assessment of the reliability of outcomes derived from such integration [11]. Furthermore, the introduction of AI into the legal field must not compromise fundamental legal principles such as fairness, transparency, accountability, and privacy. Although legal frameworks vary significantly across jurisdictions, these core principles remain central to AI governance worldwide. These foundational concerns transcend jurisdictional boundaries and provide a common basis for assessing AI adoption in legal contexts.

Thus, this paper aims to explore the importance of adopting AI within a legal framework, identifying key challenges encountered during its integration into legal practices. While regulatory frameworks differ across jurisdictions, fundamental AI adoption challenges—such as liability, transparency, privacy, and bias—are widely recognized across legal systems. This study does not focus on jurisdiction-specific regulations but rather examines broadly applicable issues that influence AI adoption in diverse legal environments. By addressing these challenges from a generalizable perspective, this study aims to provide insights that remain relevant across different legal contexts.

Current research reveals fundamental principles and challenges associated with AI, as identified by studies conducted by Felzmann et al. [12], Ferrer et al. [13], Kingston [14], and von Eschenbach [15]. Furthermore, the opportunities and inherent challenges of AI have been illuminated from diverse perspectives through the work of Dwivedi et al. [16], Lockey et al. [17], Robles Carrillo [18], Rodrigues [19], Vesnic-Alujevic et al. [20]. Research on the intersection of AI and legal domains highlights the growing integration of AI technologies into legal frameworks, a trend underscored by Surden [21]. Moreover, empirical studies and case analyses regarding AI implementation in legal contexts shed light on the practical applications of AI technologies in supporting legal decision-making processes, as demonstrated by Greenleaf et al. [22], Sourdin [23], Stevens et al. [24].

Meanwhile, the evolving landscape of AI within the legal profession has prompted critical reflections on the rapid integration of technology. Concerns have been raised about the premature reliance on technology, emphasizing the vital need to thoroughly consider ethical implications. This caution suggests a deeper exploration of how AI interacts with ethical standards within legal practices [25].

Furthermore, the ethical, legal, and social impacts of incorporating AI into the judicial domain have been significantly underscored, highlighting the complex challenges and considerations that arise when blending advanced technology with traditional legal frameworks [26]. The influence of AI on law firms has been analyzed across multiple dimensions, including business operations, models, and organizational structures. This comprehensive exploration suggests a profound transformation within the legal industry, driven by the capabilities of AI to reshape existing practices and introduce novel approaches to legal services [9].

Concurrently, the implementation of AI in legal settings has created the necessity for robust legal regulations. Such regulations aim to ensure digital governance by emphasizing transparency, accountability, and responsible management of learning algorithms, thereby addressing potential challenges in the regulation of AI technologies [27]. Moreover, exploring the potential limitations of AI in legal practice reveals that while AI can perform various scientific tasks, it may not fully comprehend the nuanced, essential first-person perspective required for moral judgments. This limitation underscores the indispensable role of human intervention in the law, advocating for a balanced approach where AI supports rather than replaces human legal judgments [28]. These insights emphasize the need for thoughtful integration of AI into the legal field, ensuring that technological advancements enhance rather than undermine the core values and ethical standards of legal practice.

Despite these extensive contributions, the existing literature still lacks a detailed examination of the practical challenges and impacts of AI within the legal sector. This highlights an urgent need for research that comprehensively addresses these unique challenges. Consequently, our investigation aims to enhance both the scholarly discourse and practical application by addressing key questions: What are the significant issues influencing the adoption of AI in the legal field, and what is the importance and ranking of each challenge? Our study represents the first comprehensive investigation into the challenges that influence AI adoption in the legal sector while also clarifying their relative importance. Rather than focusing on the specificities of any one legal system, our analysis identifies challenges that are broadly relevant across jurisdictions. The hierarchical framework developed in this study provides a structured approach to understanding AI adoption challenges in a way that is adaptable to diverse legal and regulatory environments. In addressing our research questions, we have made several contributions. First, we identify critical challenges related to AI adoption in the legal sector from the existing literature. These issues are meticulously defined, and their influence on integrating AI into legal processes is articulated. We then differentiate these concerns into various aspects and develop a hierarchical model to systematically categorize them. Employing the Analytic Hierarchy Process (AHP), we leverage the expertise of professionals in the legal domain to assess the importance and ranking of these issues, resulting in a prioritized list. This rigorous approach allows us to discern the nuances and gravity of each issue, thereby providing a comprehensive roadmap for navigating the intricate journey of AI integration in legal practices. This article is structured as follows. In the next section, an overview of the literature review is provided, focusing on the background of AI in the legal field and the primary challenges of adoption within the legal sector. Section 3 explains our research methodology and the study’s flow, while Section 4 presents the results. In Section 5, we discuss the implications of the research findings, including insights derived from the study results, limitations encountered, and future research opportunities. Finally, the conclusion provides closing remarks on the study.

## 2. Literature review

### 2.1. AI in the legal domain

In the legal field, the application of AI involves using computer and mathematical techniques to make the law more comprehensible, manageable, useful, accessible, or predictable [21]. In a broader context, the application of various technologies, including AI, to the legal field is sometimes referred to as “legaltech” or “regtech” [29]. Discussions surrounding AI adoption in the legal domain predominantly focus on practical areas [30]. The intersection of the legal field and AI began with the idea that AI could perform legal reasoning processes [10]. While early AI systems, such as rule-based expert systems like TAXMAN [31], were among the first attempts to integrate AI into legal reasoning, this paper focuses on the more recent advancements in AI technologies that have been driven by developments in machine learning, natural language processing, and predictive analytics. These modern technologies have unveiled the potential for AI adoption and utilization in the legal field, enhancing efficiency and accuracy. Moreover, studies suggest that AI adoption in legal services enhances access to justice, reduces operational costs, and facilitates more informed decision-making, underscoring its transformative potential [26,60]. This trend is expected to continue, with AI being increasingly applied in everyday tasks across all levels, ultimately transforming the legal landscape [32].

AI in the legal domain can broadly be categorized into three key areas: *(1) document analysis, (2) legal research, and (3) prediction and judgment*. Within the broad field of document analysis, various subcategories include contract analysis, document review, e-discovery, and due diligence [33]. AI-driven solutions have markedly improved efficiency in legal workflows by automating repetitive tasks, minimizing human errors, and expediting legal processing times [9]. For instance, JPMorgan successfully reduced its annual contract review time by 360,000 hours by utilizing its proprietary program, Contract Intelligence, affectionately known as “COIN” [32]. This case exemplifies how AI-driven automation streamlines legal workflows and boosts productivity, showcasing its concrete benefits in legal practice.

Legal research involves a broad spectrum of analyses and predictions, with software applications like Westlaw and LexisNexis falling into this category [34]. They provide litigation analysis tools that analyze case data and other relevant information [35]. Research indicates that AI-powered legal research tools allow lawyers to efficiently retrieve and analyze vast amounts of case law and legal precedents, streamlining legal inquiries and enhancing the accuracy of case assessments [26,60].

The area of prediction and judgment involves utilizing tools such as COMPAS for recidivism prediction. Such tools share the common goal of enhancing efficiency in performing tasks with improved accuracy and effectiveness [8]. Predictive analytics has increasingly been applied in the legal field to enhance decision-making, equipping judges and legal practitioners with data-driven insights for risk assessment and case outcome predictions [60]. As part of ensuring these improvements, AI adoption in the legal field often emphasizes the use of proven and validated technologies [36].

The advancement of AI systems, driven by progress in machine learning and natural language processing capabilities, has enabled the analysis of complex datasets. Machine learning algorithms exhibit a form of “self-learning” and can, to some extent, emulate the functionalities of the human brain [37,38]. As the time required for process-centric tasks diminishes, there is an increasing likelihood that AI may replace jobs in the legal field. This prospect has raised concerns due to the exponential growth and innovation in computing power, as well as the promised efficiency, and speed [30]. However, it is essential to acknowledge that current AI capabilities are limited, preventing them from replacing legal professionals such as lawyers who engage in intricate tasks. AI systems lack the essential capability of creative thinking, which is crucial for problem-solving [39]. Consequently, while the overall impact of AI on the legal profession remains complex and context-dependent, its capabilities continue to evolve, enabling its integration into various aspects of legal practice [21,25].

Building on this steady progress, AI will play an increasingly integral role in legal services, complementing rather than replacing human expertise [23]. As AI technologies continue to evolve, they are poised to refine key areas such as contract automation, dispute resolution, and legal risk assessment. Recognizing both the opportunities and challenges of AI adoption is essential for effectively integrating these technologies into legal practice.

### 2.2. Challenges of AI adoption in the legal domain

#### 2.2.1. Transparency.

The lack of transparency in AI systems represents a significant issue in the adoption and utilization of AI. Transparency is closely linked to the way AI systems make decisions [21,25]. The complexity of AI systems and the challenge of explaining them often lead to their perception as “black boxes” [40,41]. The characteristic of this black box, which highlights a clear absence of causal mechanisms for producing specific outputs, emphasizes the need for transparency in AI algorithms. Most of the data, processes, or outcomes used in the majority of AI algorithms are kept proprietary, making in-depth investigation challenging [42]. The absence of transparency is considered a significant flaw in ensuring the safe application of AI [34]. Conversely, transparency in AI systems has been shown to have a positive impact on trust [43–45]. In the context of adopting AI systems in the legal field, transparency plays a crucial role in preventing unfairness and the misuse of justice [46].

#### 2.2.2. Liability.

One of the major challenges in integrating AI into the legal sector is liability. In the legal context, liability is determined by the potential harm to life and property that may arise from the implementation and utilization of AI systems [19]. A longstanding fundamental question has been who should bear responsibility when damage occurs due to a flaw in an AI system [47]. The feasibility of imposing criminal liability related to AI systems has been explored [14]. Particularly within the legal domain, such liability issues require careful consideration. Assigning liability can be relatively straightforward when identifiable entities are involved in the decision-making process; however, AI systems often involve a range of parties, including data providers, designers, manufacturers, programmers, users, and the AI system itself. In such instances, clearly ascertaining liability when problems occur becomes challenging [19]. In the legal field, liability issues must be handled with great sensitivity and clarity. This involves not only determining responsibility or damages for wrongful acts but also the complex task of identifying and assigning liability [48]. Especially in the realm of law enforcement, there is an increasing inclination to outsource parts of the decision-making process to technological tools. Consequently, there is a trend towards assigning responsibility for the decision-making process to these technological tools [49].

#### 2.2.3. Bias and discrimination.

Just like human actors, AI can exhibit bias and make discriminatory judgments in the decision-making process, whether intentionally or unintentionally [50]. The outcomes of judgments and decisions generated by AI may exhibit types of bias and discrimination that society deems undesirable [51]. Particularly in the legal domain, the biases and discrimination inherent in AI warrant attention and vigilance [21]. The sources of bias in AI systems can be traced back to the data and algorithms [42]. Various studies have classified the types of biases that can influence AI systems and identified their sources [13,52]. AI based on machine learning learns models that extract specific rules from provided data. During this process, biases inherent in the data itself or the designed algorithms may exist [52]. Consequently, machine learning models trained on such data or AI systems based on these models can exhibit similar biases. Furthermore, AI algorithms are applied across various fields and domains, potentially reinforcing existing biases. In other words, “biased input leads to biased output” [53]. The severity of this issue is exacerbated by these biases, not only reflecting existing social discriminations but also amplifying and perpetuating them [54]. An appropriate analogy for the harm caused by AI decision-making might be that it represents not intentional discrimination but an unjustifiable contamination of society [55].

#### 2.2.4. Regulation.

Policymakers are concerned that without adequate regulation, insufficient trust in AI could lead to missing out on its benefits, while excessive trust could create problems [56]. These concerns arise from the rapid development and increasing use of AI technologies, sparking worries about the need to prevent negative consequences and potential harm. The regulatory challenge in this context affects the governance and control of AI technologies, making it complex and fraught with problems [57,58]. The absence of an appropriate regulatory framework can lead to difficulties and uncertainties in the adoption and use of AI technologies [59]. Legal regulations become even more crucial, especially when AI is introduced into legal domains where it could infringe upon the fundamental rights of individuals or society [60]. The legal field is influenced by a complex system of international laws, various regulations, and norms. These legal and regulatory frameworks play a positive role in mitigating risks and uncertainties while also acting as constraints by ensuring compliance [61]. In the legal domain of AI technology adoption, the main considerations revolve around safety and reliability. Establishing a regulatory framework that ensures the outcomes of AI adoption are reasonable and accurate, in accordance with established standards and rules, is critical to achieving this goal.

#### 2.2.5. Privacy.

In the legal sector, the integration of AI raises profound privacy concerns with societal and ethical dimensions. As legal professionals utilize AI to handle sensitive client data, maintaining privacy becomes paramount. This necessitates strict compliance with data protection laws to respect individuals’ privacy rights [62]. The gravity of this challenge lies not only in unauthorized data usage but also in the potential erosion of client trust, given the capacity of AI for extensive data analysis and profiling without transparent consent mechanisms [19]. This situation is exacerbated by the potential for AI systems to facilitate surveillance and unauthorized interference in private matters, which are viewed as significant violations of personal autonomy and societal privacy norms [17,63]. Legal institutions are compelled to ensure that AI applications align with ethical standards. The processing of personal information must adhere to legal statutes like GDPR, reinforcing accountability and protecting privacy as a fundamental societal value [64,65].

#### 2.2.6. Security.

From a technical perspective, the security implications of implementing AI in the legal sector are crucial. The reliance of AI systems on extensive data repositories for legal analysis makes them vulnerable to cyber threats that could jeopardize client confidentiality and the integrity of legal proceedings [66]. The sophistication of these threats necessitates advanced cybersecurity strategies to protect network resources and safeguard sensitive legal data against unauthorized access, breaches, and data manipulation [19,67]. This complexity is further heightened by the legal sector’s obligation to preserve the confidentiality of client information, which is integral to the operation of the justice system. Implementing robust cybersecurity measures, such as encryption and access control, is vital to prevent the illicit alteration of AI-driven legal decisions or the leakage of proprietary algorithms. Such breaches could lead to economic losses and compromise legal integrity [68,69]. Addressing these technical challenges is essential for the legal sector to harness the benefits of AI while upholding its duty to secure justice and protect the rights of individuals in the digital age.

#### 2.2.7. Domain knowledge.

The domain knowledge problem in legal AI arises from AI’s inherent difficulty in comprehending the intricacies of legal operations and nuances. For example, legal expert systems, which are designed to replicate the decision-making processes of human lawyers, encounter the challenge of accurately embodying complex legal rules and reasoning [70]. These systems rely on legal experts, who are often scarce, particularly those skilled in both law and computational methods [71]. The nature of law as a flexible and evolving construct, subject to varied interpretations and constant updates, exacerbates this issue, making it difficult for AI to adapt swiftly to new legal developments [23,72]. To mitigate these challenges, sustained commitment from legal experts to continuously integrate current and comprehensive legal knowledge into AI systems is required [72].

#### 2.2.8. Complexity and technical expertise.

On the technical side, the complexity and technical expertise required in legal AI are evident in the significant challenges associated with the design, development, and implementation of AI in the legal field. Legal professionals often lack the advanced technical skills required to operate and manage AI systems, which necessitate a profound understanding of machine learning algorithms, data science, and software engineering [13]. Furthermore, the labor-intensive process of labeling data for AI in law demands costly expert input, which can hinder widespread adoption [9]. This complexity is not only a barrier to the development of AI but also to its integration with existing legal processes, where AI must align with intricate legal workflows and data systems [67]. Addressing these complexities requires a concerted effort to improve the technical skills of legal professionals and to streamline AI tools for easier accessibility and integration within the legal ecosystem.

#### 2.2.9. Public acceptance.

Public acceptance of AI in the legal sector significantly depends on societal trust, which is deeply rooted in understanding AI’s commitment to the values of justice and fairness inherent in law [48]. Acceptance is cultivated through the demonstration of AI’s ethical applications and its support of legal professionals, ensuring that the technology aids rather than replaces human expertise, and enhances the accessibility and effectiveness of legal services [9]. Trust in legal AI also develops from direct, positive experiences where AI tools consistently uphold the law, respecting the nuanced traditions of the legal system [23]. Crucial to this acceptance is the assurance that AI applications serve the public interest and honor the dignity of the legal profession [73]. Involving legal experts and the general public in the development phase of AI can strengthen this trust, positioning AI as an ally in the quest for justice. Public dialogue about AI in the legal domain, emphasizing its potential to enhance rather than disrupt legal processes, will also promote greater acceptance [63].

#### 2.2.10. Data quality.

In the legal field, data quality is paramount for AI systems to make accurate and fair decisions. The integrity and reliability of data directly influence the performance of AI. Any compromise in data quality can lead to incorrect conclusions and undermine the efficacy and trustworthiness of the system [67]. Effective data governance is crucial to ensure that the data driving AI technologies are of high quality, especially in law, where decisions can have profound implications on individuals’ rights and liberties [64]. The challenge lies not only in gathering enough data but also in ensuring its consistency, validity, and integrity across diverse legal contexts. Addressing these data quality issues requires rigorous methodologies for data collection, processing, and analysis, alongside strict governance to maintain the standards necessary for the ethical and just application of AI in legal practices [13,63].

#### 2.2.11. Intellectual property.

The acceleration of AI in the legal domain brings complex intellectual property issues to the forefront. Traditional intellectual property systems are being reevaluated due to AI’s ability to autonomously generate inventions and creative works, as highlighted by [74,75]. These issues include the ownership of AI-generated content, whether such creations qualify as prior art, the management of datasets for AI learning, and accountability for AI’s creative outputs that might violate existing rights [19]. As AI becomes proficient at generating original works, determining rightful ownership poses a growing challenge. The management and ownership of datasets crucial for AI development highlight significant intellectual property concerns, especially as these systems require extensive data for training [76]. Therefore, revisiting traditional intellectual property concepts such as copyright, patent, and trademark rights, and developing a future-oriented solution through a global, multidisciplinary approach is imperative. Adapting legal frameworks to foster innovation while safeguarding intellectual property rights is crucial to address the legal challenges of AI’s evolution [19,74–76].

## 3. Methodology

This study follows a structured methodology comprising two key phases: a systematic literature review based on the PRISMA guideline and the Analytic Hierarchy Process (AHP). The systematic review identifies the key challenges associated with AI adoption in the legal domain, while the AHP evaluates and ranks these challenges based on their significance.

### 3.1. Systematic review to identify key challenges

The purpose of this systematic review is to comprehensively examine existing literature to identify the primary challenges impacting the adoption of AI in the legal field. By focusing on barriers specific to legal contexts, this review aims to provide a structured understanding of the obstacles to AI integration, forming a foundational basis for the subsequent prioritization and evaluation of these challenges.

To achieve this, the systematic review addresses the following research questions:

1. *What specific barriers or challenges are recognized in existing literature as hindering the adoption of AI within the legal domain?*
2. *How can these identified challenges be systematically organized to enhance understanding of their impact on AI adoption?*

The results of this systematic review answer these questions by providing a clear definition of each challenge and categorizing them to highlight distinct dimensions within the context of legal AI adoption. This organized framework establishes a foundation for the prioritization phase, where each identified challenge will be evaluated for its relative importance. The review was conducted following the PRISMA guidelines, ensuring that the process is transparent, replicable, and comprehensive in its approach to literature selection and analysis across multiple disciplines, including legal studies, computer science, and social sciences (S4 File).

#### 3.1.1. Search strategy.

To capture a comprehensive range of literature on challenges associated with AI adoption in the legal field, a systematic search was conducted in alignment with PRISMA guidelines. This search aimed to include studies from multiple disciplines to ensure an interdisciplinary understanding of the barriers impacting AI integration in legal contexts. We utilized Web of Science, IEEE Xplore, Westlaw, and Google Scholar, selected for their relevance to law, technology, and social sciences.

The search was conducted from April to May 2024. Specific keywords were selected to cover a broad range of AI-related terminology, adoption contexts, and legal applications. The following search string was applied consistently across all databases:

(“Artificial intelligence” OR “AI” OR “Machine learning” OR “Deep Learning” OR “Natural Language Processing” OR “NLP”) AND (“Adoption” OR “Implementation” OR “Integration” OR “Utilisation” OR “Application” OR “Deployment”) AND (“Law” OR “Legal” OR “Judicial” OR “Court” OR “Legal System” OR “Justice System”) AND (“Challenge” OR “Obstacle” OR “Barrier” OR “Issue” OR “Difficulty” OR “Problem” OR “Risk” OR “Limitation”).

This approach ensured a systematic and comprehensive literature retrieval, capturing a wide array of studies addressing diverse aspects of AI adoption challenges within legal contexts.

#### 3.1.2. Eligibility criteria.

To ensure the relevance and quality of the studies included in this review, specific eligibility criteria were applied. Only peer-reviewed articles published in English were included to maintain consistency and academic rigor. Additionally, studies published from the year 2000 onwards were considered to reflect recent advancements in AI within the legal field.

In terms of topical relevance, studies had to address the adoption, implementation, or integration of AI within legal contexts to be prioritized for inclusion. Studies focusing on AI applications outside the legal domain, such as in healthcare or finance, were excluded unless they provided transferable insights applicable to legal contexts. Furthermore, articles that discussed AI adoption in broad terms without addressing specific challenges within the legal field were excluded, as well as studies lacking detailed discussions or well-supported analyses of AI adoption challenges in the legal field.

These criteria were applied systematically to refine the search results, ensuring that the final set of articles aligns closely with the review’s objective of identifying significant barriers to AI adoption in legal contexts.

#### 3.1.3. Bias and quality assessment.

To ensure rigor and reliability, both the Risk of Bias in Systematic Reviews (ROBIS) tool [77] and the JBI Critical Appraisal Checklist for Qualitative Research [78] were applied. The ROBIS tool assessed bias in three phases: *(1) relevance, (2) review process concerns, and (3) overall risk of bias*. Two reviewers (SK and SY) independently evaluated bias across four domains, resolving any discrepancies through discussion, with a third reviewer (SP) as needed. The ROBIS assessment indicated a low to moderate overall risk of bias, supporting the reliability of the findings.

Additionally, the JBI Critical Appraisal Checklist evaluated the quality of included qualitative studies, focusing on methodological congruity, researcher influence, and ethical considerations (S2 File). This assessment further strengthened the review by confirming that most studies met high-quality standards in key areas.

#### 3.1.4. Results.

**Fig 1:**
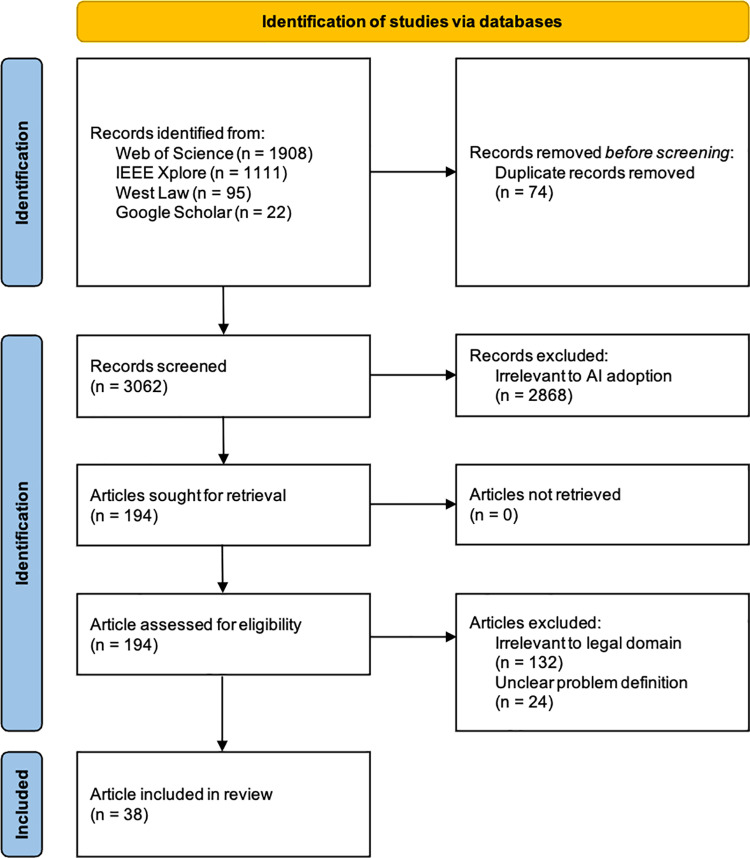
PRISMA flow diagram.

The initial search yielded 3,136 articles, with 74 duplicates removed. The remaining 3,062 articles underwent title and abstract screening, leading to the exclusion of 2,868 studies that lacked relevance to AI adoption issues. After full-text review of the remaining 194 studies, an additional 156 were excluded due to irrelevance to the legal domain or unclear problem definitions. A final selection of 38 articles was approved by the three authors in collaboration with external experts (S1 File). Fig 1 provides a summary of the study selection process via a PRISMA flow diagram.

As a result of this systematic review, eleven key challenges related to AI adoption in the legal domain were identified. These challenges were categorized into Legal Aspects, Technical Aspects, and Societal & Ethical Aspects to facilitate structured understanding. Challenges include issues such as Liability, Regulatory Compliance, Intellectual Property, Data Quality, Algorithmic Transparency, Fairness, and Privacy. Table 1 provides an overview of these challenges, along with their definitions and relevant references, forming the foundation for the subsequent AHP analysis.

**Table 1:** Overview of challenges identified in the literature review.

Category	Challenges	Challenges definition	References
Legal aspects	*Liability*	The legal responsibility for harm or damage caused by AI systems	[2,14,19,48,79]
	*Regulation*	The legal and regulatory frameworks governing the development and deployment of AI systems	[2,27,56,60,76,79,80]
	*Domain knowledge*	The specialized legal expertise required for AI to accurately interpret and apply legal principles within specific legal contexts	[23,72]
	*Intellectual property*	The legal rights and considerations surrounding the ownership, protection, and use of content or inventions generated or influenced by AI	[19,74,81]
Technical aspects	*Complexity and Technical expertise*	The advanced technical skills needed by legal professionals to effectively design, implement, and manage AI systems	[39,63,67]
	*Security*	The protection of AI systems and legal data from unauthorized access and cyber threats	[19,60,64,66,67,82–84]
	*Data quality*	The accuracy and reliability of data utilized by AI systems	[64,63,67,85]
Societal & Ethical aspects	*Public acceptance*	The level of societal trust and acceptance of AI within the legal field	[23,48,63]
	*Bias and discrimination*	The potential for AI systems to produce unfair outcomes by replicating or amplifying biases in data or algorithms	[13,19,21,23,33,34,54,84–89]
	*Transparency*	The clarity and openness of AI decision-making processes, essential for ensuring accountability and trust in legal contexts	[12,19,21,25,34,48,86,90,91,92]
	*Privacy*	The protection of personal and sensitive information processed by AI systems, ensuring compliance with legal standards and safeguarding individual rights	[17,19,34,60,63,64,83–87]

### 3.2. Analytic hierarchy process to evaluate key challenges

The Analytic Hierarchy Process (AHP) is a method designed for the quantitative transformation of conceptual and subjective factors to evaluate alternatives [93–95]. We utilized the AHP to quantify and calculate the relative importance of challenges extracted and identified through literature review and expert validation. This helps scholars and practitioners recognize and rank the challenges presented. The AHP has been applied in various industries and fields, including healthcare [96], manufacturing [97], automobile [98], construction management [99], and green supply chain management [100]. Studies have also utilized the AHP method in the context of AI adoption [67,101–103].

Data were gathered and analyzed from eight experts through surveys and interviews. While the sample size might appear small, AHP, unlike other methods, does not require a large sample for reliable results. Scholars argue that a larger sample size can lead to higher levels of inconsistency, making it impractical. They assert that even a small sample size is sufficient for using the AHP [99,102,104]. Various studies have successfully evaluated factors with small samples [67,102,105,106]. Prior research has shown that expert panels in AHP studies typically range from 5 to 15 participants. Such an approach maintains the reliability of expert judgments while minimizing inconsistencies that may arise in larger groups [102,104].

To achieve a comprehensive evaluation, we implemented a purposive sampling approach, selecting experts from key sectors relevant to AI adoption in the legal domain, such as academia, law firms, courts, corporations, and regulatory bodies. This multidisciplinary approach ensures comprehensive coverage of key perspectives in AI adoption. All selected experts have over a decade of experience in AI-related legal practice or research. Their extensive background ensures that our evaluation incorporates both theoretical insights and practical considerations, resulting in a rigorous and professionally relevant assessment.

Table 2 presents the demographic details of the experts who took part in this study. The experts comprise professors, a judge, lawyers, a patent attorney, a regulatory compliance specialist, and an AI engineer. They work in research institutions, courts, law firms, and corporations, bringing extensive experience in AI projects, with a strong focus on practical applications in the legal field. Their diverse backgrounds offer a comprehensive perspective on key stakeholders in AI adoption in the legal sector. Approximately 63% of the experts hold master’s degrees, and 25% possess doctoral degrees.

**Table 2:** Participants’ demographic profile.

Demographic		Frequency	Percentage
Gender	Male	6	75%
	Female	2	25%
Age	30-40	4	50%
	40-50	3	37.5%
	50-60	1	12.5%
Education	Bachelor	1	12.5%
	Master	5	62.5%
	Doctoral	2	25%
Experience	10-15 years	5	62.5%
	15-20 years	2	25%
	> 20 years	1	12.5%
Occupation	Professor	2	25%
	Judge	1	12.5%
	Lawyer	2	25%
	Patent attorney	1	12.5%
	Regulatory compliance specialist	1	12.5%
	AI engineer	1	12.5%

All experts voluntarily participated in the study after being fully informed of its purpose, scope, and procedures. The expert survey was conducted between May 8, 2024 and May 31, 2024. This study was conducted as part of a broader research project approved by the Institutional Review Board of KAIST (Korea Advanced Institute of Science and Technology) on December 5, 2023 (IRB approval number: KH2023−247). Written informed consent was obtained from all participants prior to data collection. No personally identifying information was collected, and all data were anonymized before analysis.

The proposed methodology involves a detailed research process using the AHP to evaluate and prioritize the challenges associated with AI adoption in the legal field. The process is structured in several stages to systematically identify, categorize, and validate these challenges.

Step 1. Identify challenges

In the first step, challenges related to the adoption of AI in the legal field are identified and extracted through a systematic literature review. Eight experts are then presented with these identified challenges, whose feedback is meticulously verified for substance, importance, and both academic and practical relevance. After consolidating the experts’ feedback, it is confirmed that these challenges are significant concerns for the adoption of AI in the legal field. The identified challenges are described in Section 2.2.

Step 2. Categorize challenges

**Fig 2:**
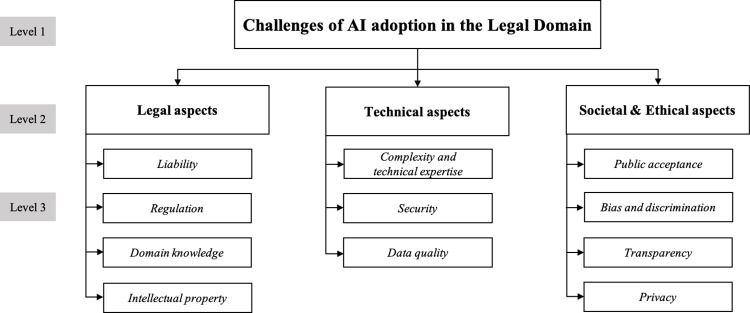
Hierarchical structure of challenges.

The second step involves categorizing the verified challenges into three significant areas: legal, technical, and socio-ethical. This classification provides a structured approach to identifying fundamental challenges influencing AI adoption, focusing on constraints that directly shape its development, implementation, and acceptance. The review process considered multiple dimensions, including legal, technical, socio-ethical, economic, and operational factors. While economic and operational concerns are significant, their impact was found to be highly dependent on external conditions such as institutional structure, market dynamics, and regulatory environments. In contrast, legal, technical, and socio-ethical challenges consistently emerged as primary determinants, shaping the core barriers and requirements for AI adoption. Expert validation further confirmed that economic and operational concerns were often discussed in relation to these broader dimensions rather than as independent categories. Thus, economic and operational aspects were incorporated within the proposed classification framework, as they are frequently influenced by legal, technical, and socio-ethical constraints. This approach ensures a structured and coherent analysis of the key challenges in AI adoption. Fig 2 illustrates the hierarchy of these categories and their associated challenges.

Step 3. Assess challenge significance

The third step involves requesting experts to assess the significance of each challenge through surveys and interviews, utilizing a comparative judgment approach. The survey used Saaty’s 9-point scale, as presented in Table 3, to compare pairs of challenges. The five odd values—1, 3, 5, 7, 9—correspond to ‘equal importance,’ ‘moderate importance,’ ‘strong importance,’ ‘very strong importance,’ and ‘extreme importance,’ respectively. For example, to compare the importance of the regulatory challenge to the transparency challenge, we asked, “How much more important is the ‘regulation’ challenge compared to the ‘transparency’ challenge in the adoption of AI in the legal field?” If an expert determines that the regulatory challenge has “very strong importance” over the transparency challenge, a value of 7 is assigned. Conversely, if the transparency challenge is considered to have “very strong importance” over the regulatory challenge, a reciprocal value of 1/7 is assigned. This same process was used for 55 pairs of challenges (S3 File).

**Table 3:** Scale measurement.

Scale	Intensity of importance	Reciprocal
Equal importance	1	1/1
Moderate importance	3	1/3
Strong importance	5	1/5
Very strong importance	7	1/7
Extreme importance	9	1/9

Given the complexity of the AHP method, we conducted preliminary expert discussions before the survey to refine variable definitions, establish shared terminology, and ensure that all experts had a common understanding of the evaluation framework. After the survey, follow-up discussions were held to align interpretations of the pairwise comparison criteria and resolve potential inconsistencies in expert judgments. These discussions were not independent data collection methods but an integral part of the AHP process, enhancing the validity and reliability of expert evaluations. By maintaining clear communication throughout these discussions, we minimized discrepancies in expert assessments and reinforced the coherence of their responses in the AHP survey.

Step 4. Analyze data

The fourth step involves data analysis. We analyzed the comparison matrix completed in the previous stage to rank the categories and challenges based on hierarchy. During this process, the measurements and weights of all challenges were calculated, and the consistency of the experts’ judgments was assessed by calculating the consistency index (CI) and consistency ratio (CR). The CI is calculated as follows:


$$\begin{array}{c} \text{CI}=\frac{\left(\lambda_{\max}-n\right)}{\left(n-1\right)} \end{array}$$

where λmax is the maximum eigenvalue of the paired comparison matrix, and n is the number of criteria. The CR is then determined by dividing the CI by the random consistency index (RI):


$$\begin{array}{c} \text{CR = CI / RI} \end{array}$$

The CI varies randomly depending on the sample size and is presented in Table 4. Saaty [94] provides criteria for verifying the consistency of judgments. A CR that does not exceed 0.1 is considered consistent. If this value is exceeded, the comparison matrix is deemed inconsistent, necessitating a review and improvement of the judgments.

**Table 4:** Random consistency index values.

Number of factors	1	2	3	4	5	6	7	8	9	10	11	12
Random consistency index	0	0	0.58	0.90	1.12	1.24	1.32	1.41	1.45	1.49	1.51	1.48

Step 5. Rank challenges

In the final stage, we confirm the ultimate ranking of challenges associated with the adoption of AI in the legal sector based on the analyzed data. By integrating the results of the data analysis and the opinions of the experts, we derive academic and practical implications.

### 3.3. Summary of the research process

**Fig 3:**
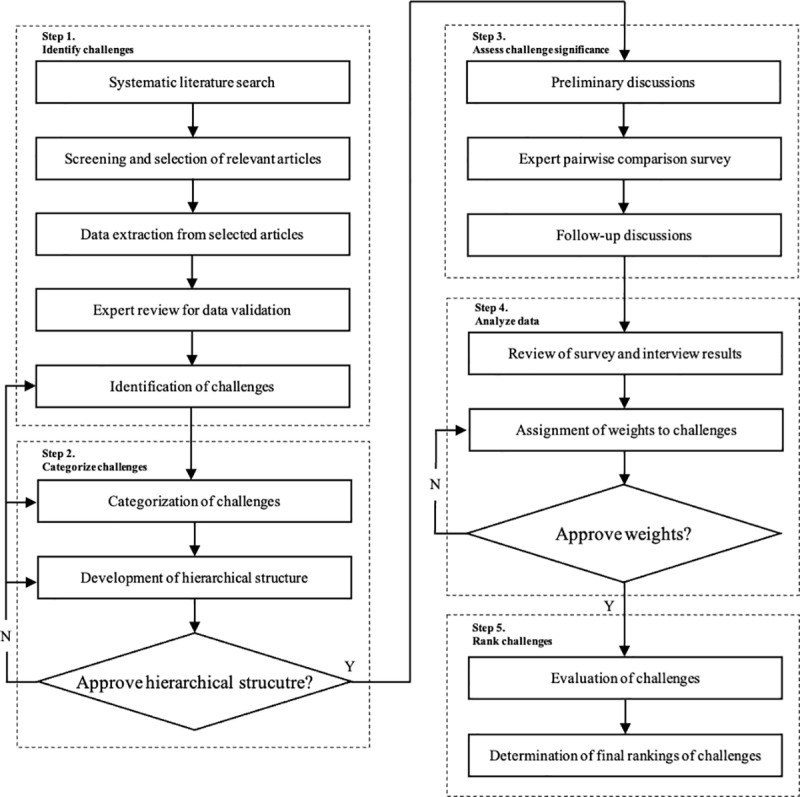
Research process flowchart.

The research process illustrated above, including the identification, categorization, and assessment of challenges, is visually summarized in Fig 3.

## 4. Results

According to the RI presented by Saaty [94], the value for a matrix with 11 challenges is 1.51, as shown in Table 4. In our study, the calculated consistency index was 0.06464, which results in a consistency ratio of 0.04281 when divided by 1.51. This ratio falls well below the acceptable threshold, indicating a satisfactory level of consistency according to the standard criteria.

Table 5 provides a focused summary of the AHP analysis, emphasizing the most critical aspects of the results:

**Table 5:** Summary of AHP priority weights and rankings.

Category	Relative weight across categories	Challenge	Relative weight within categories	Ranking	Relative weight among challenges	Ranking
**Legal aspect**	0.38	Liability	0.53742	1	0.22550	1
		Regulation	0.26144	2	0.10969	4
		Domain knowledge	0.10200	3	0.04282	10
		Intellectual property	0.09914	4	0.04160	11
**Technical aspect**	0.29	Complexity and technical expertise	0.21537	3	0.05101	9
		Security	0.23649	2	0.05599	8
		Data quality	0.54814	1	0.12978	2
**Societal** **& Ethical aspect**	0.31	Public acceptance	0.22526	3	0.07739	6
		Bias and discrimination	0.24127	2	0.08287	5
		Transparency	0.34168	1	0.11742	3
		Privacy	0.19179	4	0.06594	7

1. Category: The first column lists the three primary categories that structure the analysis: Legal aspect, Technical aspect, and Socio-ethical (i.e., “Societal and Ethical”) aspect. These categories provide the framework within which the specific challenges are evaluated.
2. Relative weight across categories: This column shows the relative importance of each category in the overall analysis. The results indicate that the Legal aspects category is the most significant, representing 38% of the total weight. This is followed by Socio-ethical aspects at 31% and Technical aspects at 29%. These weights highlight the dominant concerns about legal implications in AI adoption while recognizing the importance of socio-ethical and technical considerations.
3. Relative weight within Categories: The relative weights of individual challenges within each category are presented in this column. For example, “Liability” holds the highest weight within the Legal aspects category, signifying its critical importance. Similarly, “Data quality” is the most significant challenge within the Technical aspects category, and “Transparency” is the leading concern in the Societal & Ethical aspects category.
4. Relative weight among challenges: This column compares the overall importance of all 11 challenges across the categories. “Liability” is the most critical challenge overall, with the highest weight across all categories. On the other hand, “Intellectual property” is deemed the least important, reflecting its relatively lower impact than other challenges.

**Fig 4:**
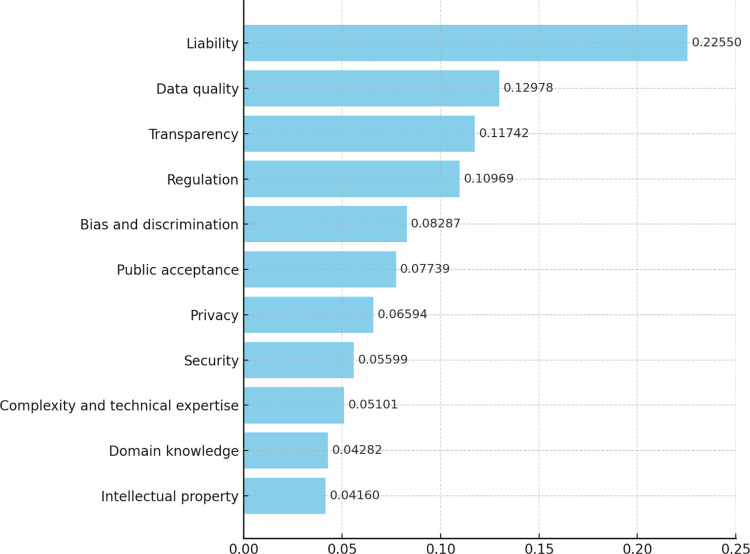
Challenges ranked by importance and their corresponding weights.

Fig 4 graphically represents the results from the fifth and sixth columns of Table 5, arranged in descending order, making it easier to identify the most and least critical challenges and providing a clear visual summary of the priority areas in the AHP analysis.

## 5. Discussion, implications, and limitations

### 5.1. Discussion

This study explores the critical challenges associated with the introduction of AI in the legal sector by collecting expert opinions to determine the importance and priority of these challenges. Through a systematic review, 11 key challenges were identified, and their significance was evaluated and confirmed by 8 legal experts with extensive experience in AI-related work and research. The results of this study are valuable for comprehending the importance of each category and the challenges within them. We identified three primary categories and 11 secondary factors, with legal aspects emerging as the most crucial category according to our findings. The legal category, which constitutes 38% of the total weight, is composed of four main factors: ‘Liability’, ‘Regulation’, ‘Domain Knowledge’, and ‘Intellectual Property’.

In our study, “Liability” was assessed as the most crucial factor, with a weight of 0.22550 among all factors. As Krausová [107] discussed, the majority of experts involved in the study emphasized their growing concern over legal issues and litigation risks that could arise due to increased automation and legal judgments. This underscores the importance of establishing clear legal accountability for decisions made by AI-based systems, which is expected to greatly influence future legislation and policy development related to AI [84,108]. These findings also suggest the necessity of developing new protocols to enhance collaboration between legal professionals and technologists and to ensure that AI system decisions align with legal standards. Thus, “liability” is a crucial challenge that necessitates careful consideration and management during the integration of AI in the legal process.

“Regulation” ranks fourth among the 11 factors. These analyses reflect the need to manage uncertainties and create a predictable legal environment when AI is adopted and utilized in legal practices. Our research acknowledges the role of regulation in highlighting the significance of AI system adoption within organizations [109] and emphasizes the need for legal regulations to address the negative impacts of AI [60,84]. Therefore, AI adoption poses new challenges to existing legal regulatory frameworks, suggesting the need for new regulatory approaches in legal technology. Regulatory bodies and legal professionals should focus on assessing the potential risks of AI and developing regulatory frameworks suited for technological advancements. These efforts are expected to lead to significant changes in various aspects of the legal field, such as the delivery of legal services, law enforcement, and fairness standards. This study contributes to laying the foundation for the proper and effective integration of AI in the legal domain.

“Domain knowledge” has been confirmed as a foundational factor for the effective functioning of AI in the legal field. Although not given high weight by experts, “Domain Knowledge” is agreed upon as playing a crucial role in providing legal judgments and recommendations by AI systems. For AI systems to accurately interpret and apply complex laws, a profound understanding of the legal field is necessary [72]. Specifically, the accurate use of legal terminology, understanding of legal context, and careful interpretation of legal regulations are impossible without the domain knowledge provided by legal professionals [23,110]. Therefore, this study emphasizes the importance of technology developers who aim to integrate AI in the legal sector to prioritize collaboration with legal experts and incorporate an understanding of legal context and principles in AI education programs.

‘Intellectual Property’ was ranked as the least important factor with the lowest weight among all factors. This suggests that the management and protection of intellectual property are relatively less crucial compared to other challenges in the adoption of AI in the legal field. Some experts have mentioned that intellectual property issues might not be perceived as an urgent practical task that needs to be addressed. In the initial stages of AI adoption, more attention should be directed at fundamental aspects such as the performance, stability, and legal compliance of the technology, with detailed management aspects like intellectual property being considered further down the adoption process. From this perspective, other factors take precedence over intellectual property in the adoption phase, which may be reflected in the weight evaluation. However, issues related to intellectual property in the context of AI use remain a significant concern that demands attention and caution [76]. This paper evaluates the challenges faced by AI under current intellectual property law. Adopting AI may change the existing intellectual property legal framework, requiring suitable legal responses [74].

The second most important category is socio-ethical, which comprises 31% of the total weight and includes four factors: ‘Public acceptance,’ ‘Bias and discrimination,’ ‘Transparency,’ and ‘Privacy.’

In the AHP analysis, ‘Transparency’ received a weight of 0.11742, ranking first in the socio-ethical aspects category and third among all factors. The issue of transparency in AI in the legal field is directly linked to the ability to understand and review the basis of AI’s decisions, which is essential for building trust in AI system processes [21]. It is necessary to understand how these decisions are made [84], especially in the legal field, where AI’s decisions can directly affect individual rights. Experts have expressed concerns that correcting errors could be challenging without ensuring transparency in AI used in the legal field, potentially resulting in cascading effects and issues.

The issue of “bias and discrimination” was identified as a crucial ethical concern in the implementation of AI in the legal sector, with a weight of 0.08287 [34]. Despite AI’s potential for fair and objective decision-making in the legal sector, bias inherent in the data and algorithms driving it can impact outcomes [19]. If the algorithmic decision-making process is based on biased data, the results risk being biased as well, leading to unfair judgments [67,84]. Experts highlight the potential for bias and discrimination stemming from algorithmic distortions caused by AI characteristics. Such distortions could infringe upon fundamental constitutional rights, such as the presumption of innocence and the right to equality. Thus, minimizing bias and discrimination in developing and applying AI systems is imperative.

‘Public acceptance’ ranks third in the socio-ethical aspects category and sixth overall, with a weight of 0.07739, indicating that public trust and acceptance play a crucial role in the successful adoption and utilization of AI in legal practices [13,63]. According to legal experts involved in the study, the legal field tends to adopt AI passively because of social backlash and resistance concerns. If there is a lack of understanding and trust in legal services, the practical application of such technology would be significantly limited. Therefore, communication and education with the public are crucial in adopting AI in the legal sector. This is essential to define AI’s potential and limitations clearly and to reduce misinformation and unnecessary fears. This will help the public understand how AI can be used to improve the quality and accessibility of legal services.

Our study results emphasize the significance of “privacy” in applying AI in the legal field. Since legal services often involve handling sensitive personal information, data protection during AI system data processing is a significant consideration [34,84] and is directly linked to trust issues [17]. When AI technologies process and analyze individual data, the confidentiality of such information must be maintained and handled according to legal procedures. Legal practices must strictly apply data protection principles in designing and implementing AI systems [13]. Experts emphasize that when privacy issues arise, the irreversible consequences necessitate adherence to data protection principles and related laws and regulations during AI adoption.

Finally, the technical aspects category emerged as significant, accounting for 29% of the total weight. It comprises three factors: ‘Complexity and technical expertise,’ ‘Security,’ and ‘Data quality.’

‘Data quality’ was evaluated with a weight of 0.12978, ranking first in the technical aspects and second overall among all factors. This result shows that successfully constructing accurate and reliable AI systems depends on high-quality data [76,111]. Particularly in the legal field, case analysis, legal document interpretation, and client consultation rely on accurate and trustworthy data. Errors or incompleteness in data can lead to incorrect legal advice or decisions, making data management and curation processes critical steps for the effective operation of AI systems. Experts emphasize that introducing AI in the legal field is not feasible without guaranteed data quality. Desired outcomes and user satisfaction can only be achieved when data quality is assured. Hence, legal practitioners must conduct continuous supervision and evaluation of data quality during the adoption of AI technology. This signifies that securing high-quality data is an essential condition for the success of AI-based legal services.

Similar to the issue of privacy, legal data involves sensitive and confidential information; therefore, AI systems must meet the highest security standards. According to the AHP analysis, “Security” was assessed by experts as an important factor in the technical aspects, with a weight of 0.23649. This indicates that the security level of AI systems improves user trust and confidence in the system [67], which is crucial as it can have significant implications on life, human security, and access to resources [19]. Effective management of security issues is essential when integrating AI into the legal sector. Security breaches can lead not only to a loss of trust but also to legal liabilities. Therefore, establishing security protocols and data protection mechanisms is crucial for the successful introduction of AI in legal practices.

The challenge of “Complexity and technical expertise” was evaluated with a weight of 0.05101, making it one of the significant technical hurdles in the adoption of AI in the legal sector, according to experts’ assessment. This underscores the importance of understanding the complexity of AI systems and the technical expertise required for legal professionals to utilize these technologies effectively [112]. For successful integration of AI in the legal domain, specialized technical skills are necessary to handle complex AI systems and provide appropriate tech solutions [38]. Thus, enhancing collaboration between technical developers and legal professionals, as well as developing educational and training programs to build the necessary technical skills and expertise, is essential [113]. Building technical capacities to respond to legal issues is necessary.

As revealed by this study, adopting AI in the legal sector goes beyond technological advancement, requiring thorough consideration in legal, social, and ethical contexts. The above-mentioned factors encompass various risks and could lead to severe problems if poorly managed. However, systematic management of these factors can significantly improve the legal sector’s quality through AI. Therefore, integrating AI in the legal sector requires a prudent approach that balances and coordinates these diverse factors. As the role of AI grows in the evolving legal environment, this research provides critical insights into the challenges and opportunities faced by legal professionals and technology developers. The successful introduction and integration of AI technology can enhance the efficiency, accessibility, and fairness of the legal sector, playing a vital role in enabling professionals to provide more equitable and inclusive services.

### 5.2. Implications

This study is pioneering in its comprehensive assessment of various challenges that affect the adoption of AI in the legal field. This collective inquiry, which has not been thoroughly addressed in existing literature, offers a quantitative evaluation of their relative significance.

Academically, this approach significantly contributes to future researchers’ deeper understanding and exploration of factors influencing the application and implementation of AI in the legal domain. Particularly, the AHP methodology proves effective in clarifying the priorities among different factors within complex decision-making processes. By enabling a systematic and numerical analysis of legal field issues, traditionally discussed in conceptual or qualitative terms, this research extends and strengthens the methodological foundation of the field. It thus provides a starting point for future researchers to explore the interactions and impact between the identified factors and conduct more in-depth studies comparing AI adoption strategies across different legal systems and cultural contexts. Additionally, this research underscores the necessity of thoroughly examining the multifaceted implications of potential changes and associated challenges brought about by AI advancements in the legal sector. Discussions on using AI in the legal field have mainly focused on legal and technical aspects. However, the findings also emphasize the importance of socio-ethical issues, such as transparency, bias, and discrimination. This will serve as critical foundational material for future studies aiming to develop new theoretical frameworks and methodologies to address potential issues that may arise when applying AI in the legal field.

Moreover, this study suggests the necessity of close collaboration between legal professionals possessing domain knowledge and developers with technical expertise to address the technical requirements of AI systems in the legal domain. Effective collaboration and educational programs are vital for the successful introduction of AI. Lastly, this study, conducted with the participation and assessment of experts who have actual experience in AI adoption in the legal sector, offers profound insights and practical value to practitioners.

### 5.3. Limitations and future directions

Despite providing a comprehensive analysis of the issues facing AI adoption in the legal sector, this study acknowledges certain limitations that open up several avenues for future research. This study focused on legal experts within a specific jurisdiction to investigate the challenges of AI adoption in the legal domain. While the common cultural and professional background of the participants helped minimize research bias, this focus limits the ability to fully capture perspectives from diverse legal and regulatory environments. However, many of the core challenges identified in this study—such as liability, transparency, privacy, and bias—are widely recognized across different legal systems. Variations in legal and regulatory approaches may influence how these challenges manifest in practice. Future research should further examine these jurisdictional differences to enhance the applicability of findings across diverse legal contexts.

Moreover, this study primarily relied on legal experts with experience in AI, which could introduce a potential bias towards an ‘insider’ perspective. This may have influenced the identification and prioritization of challenges, potentially overemphasizing issues familiar to these experts while underrepresenting challenges more apparent to those without AI expertise. Future research should incorporate perspectives from legal professionals who do not specialize in AI, as their insights could provide a more balanced understanding of challenges, particularly in areas such as Privacy and Public Acceptance.

Additionally, while our study focused on 11 key issues, there may be other important factors not included due to the lack of existing literature. Future studies should seek to identify additional factors through the broad participation of researchers and practitioners. Comparative legal analyses examining how different jurisdictions regulate and implement AI-driven legal technologies could provide valuable insights into the adaptability of our findings. Cross-jurisdictional case studies may further clarify the extent to which variations in legal and regulatory frameworks influence AI adoption challenges in legal practice. This process will enable the development of more precise guidelines for the application of AI technologies in the legal domain.

Our study utilized a systematic review combined with the AHP methodology to assess critical challenges associated with the integration of AI in the legal field. However, due to methodological limitations, there could have been constraints in objectively assessing all issues associated with AI adoption. This research was conducted based on thorough planning and expert opinions. The accuracy of the research findings was enhanced by analyzing data obtained through the systematic review and the AHP process. Nevertheless, additional research is needed to understand the various facets and complexities of AI adoption more deeply and objectively. While much of the existing research has primarily examined the challenges associated with AI implementation, a complementary approach is necessary to investigate its potential benefits and successful applications.

In this regard, future studies should also explore successful case studies and best practices in AI adoption. Investigating how AI has been effectively integrated into legal workflows, the conditions under which it leads to optimal outcomes, and the strategies used to mitigate risks can provide valuable insights for both practitioners and policymakers. Such research would not only contribute to a more balanced understanding of AI adoption but also facilitate the development of guidelines that maximize its benefits while addressing regulatory and ethical concerns. For example, empirical research using an expanded sample size and diverse data sources could investigate the causal impacts and intricate aspects of AI adoption in the legal field. Further exploration of successful case studies would complement this approach, offering a more comprehensive understanding of both the risks and rewards of AI implementation. This could enhance the objectivity and reliability of the research findings.

## 6. Conclusion

The integration of AI into the legal domain is not merely an option but a compelling necessity driven by contemporary societal demands. The effectiveness and value of AI in this context depend on its integration and utilization. This study aims to identify and evaluate the key challenges influencing the adoption of AI in the legal sector, thereby recognizing the primary hurdles associated with its integration. Through an extensive and systematic literature review, a wide range of factors related to AI in the legal domain were identified. Additionally, data collected from eight experts were utilized to classify, assess, and rank these challenges. By utilizing the AHP, this study thoroughly examined challenges in the legal field that were previously discussed mainly in conceptual or qualitative terms, quantitatively assessing their relative importance. According to the findings, the legal aspects emerged as the most crucial category, with legal liability being identified as the most critical challenge. These results offer practical guidelines for implementing AI in the legal field. Furthermore, our study underscores the multifaceted nature of these challenges and emphasizes the necessity for close collaboration between legal experts and technologists, along with ongoing education initiatives.

## Supporting information

S1 File Reviewed studies with inclusion and exclusion criteria. List of studies identified in the systematic review, including inclusion and exclusion criteria.
(XLSX)

S2 File JBI qualitative study assessment. Quality assessment results of qualitative studies using the JBI checklist.
(DOCX)

S3 File AHP expert judgment data. Raw expert judgment data from the AHP analysis, including pairwise comparisons.
(XLSX)

S4 File PRISMA 2020 checklist. Completed PRISMA checklist for the systematic review.
(DOCX)
