# Supplementary material for: Prioritizing challenges in AI adoption for the legal domain: A systematic review and expert-driven AHP analysis
Source: PLoS One. 2025 Jun 24;20(6):e0326028. doi: 10.1371/journal.pone.0326028 (PMC12186909; doi:10.1371/journal.pone.0326028)
Supplement: S2 File — Quality assessment results of qualitative studies using the JBI checklist. (DOCX) [file pone.0326028.s002.docx]

Quality Assessment of Included Studies Using the JBI Critical Appraisal Checklist for Qualitative Research

| **Study** | **Q1** | **Q2** | **Q3** | **Q4** | **Q5** | **Q6** | **Q7** | **Q8** | **Q9** | **Q10** |
| --- | --- | --- | --- | --- | --- | --- | --- | --- | --- | --- |
| Baker, 2018 | Y | Y | U | Y | Y | N | U | N/A | U | Y |
| Brooks et al., 2020 | Y | Y | Y | Y | Y | U | U | Y | U | Y |
| Davis, 2020 | Y | Y | N/A | Y | Y | N | N | N/A | U | Y |
| Dimyadi et al., 2019 | Y | Y | Y | Y | Y | N | N | N/A | U | Y |
| Doshi-Velez et al., 2019 | Y | Y | N/A | Y | Y | N | N | N/A | N/A | Y |
| Dulka, 2022 | Y | Y | Y | Y | Y | U | U | N/A | Y | Y |
| Felzmann et al., 2019 | Y | Y | Y | Y | Y | Y | N | N/A | N/A | Y |
| Ferrer et al., 2021 | Y | Y | Y | Y | Y | N | N | N/A | N/A | Y |
| Goodman, 2019 | Y | Y | Y | Y | Y | U | N | N/A | N/A | Y |
| Grimm et al., 2021 | Y | Y | Y | Y | Y | N | U | N/A | Y | Y |
| Hoffmann-Riem, 2020 | U | Y | N/A | Y | Y | N | U | N/A | Y | Y |
| Kaminski, 2022 | Y | Y | N/A | Y | Y | U | N | N/A | Y | Y |
| Kamble, 2024 | Y | Y | Y | Y | Y | N | U | N/A | N/A | Y |
| Kingston, 2016 | U | Y | N/A | Y | Y | N | N | N/A | U | Y |
| Kop, 2019 | U | Y | U | Y | Y | N | N | N/A | Y | Y |
| Larsson & Heintz, 2020 | Y | Y | Y | Y | Y | Y | U | N/A | N/A | Y |
| Legg & Bell, 2019 | Y | Y | Y | Y | Y | U | N | N/A | N/A | Y |
| Lockey et al., 2021 | Y | Y | Y | Y | Y | Y | U | N/A | N/A | Y |
| Madan & Ashok, 2023 | Y | Y | Y | Y | Y | Y | N | N/A | Y | Y |
| Merhi, 2023 | Y | Y | Y | Y | Y | U | N | Y | U | Y |
| Ntoutsi et al., 2020 | Y | Y | Y | Y | Y | N | N | N/A | U | Y |
| Oguama, 2022 | U | Y | N/A | Y | Y | N | N | N/A | N/A | Y |
| Rajendra & Thuraisingam, 2022 | Y | Y | Y | Y | Y | N | N | N/A | Y | Y |
| Rodrigues, 2020 | Y | Y | Y | Y | Y | Y | N | N/A | N/A | Y |
| Rodriguez, 2023 | Y | Y | Y | Y | Y | U | N | N/A | Y | Y |
| Schneider, 2022 | Y | Y | U | Y | Y | N | U | N/A | Y | Y |
| Semmler & Rose, 2017 | Y | Y | Y | Y | Y | N | U | N/A | N/A | Y |
| Sourdin, 2018 | Y | Y | Y | Y | Y | U | N | N/A | N/A | Y |
| Stoica et al., 2017 | U | Y | N/A | Y | Y | N | N | N/A | Y | Y |
| Surden, 2019 | Y | Y | N/A | N/A | Y | N | N | N/A | N/A | Y |
| Tamò-Larrieux et al., 2024 | Y | Y | N/A | Y | Y | Y | U | N/A | N/A | Y |
| Walters & Novak, 2021 | Y | Y | Y | Y | Y | N | N | N/A | U | Y |
| Webley, 2019 | U | Y | U | Y | Y | N | N | N/A | N/A | Y |
| Wirtz et al., 2019 | Y | Y | Y | Y | Y | N | N | N/A | U | Y |
| Yan, 2023 | Y | Y | Y | Y | Y | U | N | N/A | N/A | Y |
| Yara et al., 2021 | Y | Y | U | Y | Y | N | N | N/A | N/A | Y |
| Yu & Alì, 2019 | Y | Y | U | Y | Y | N | N | N/A | U | Y |
| Zodi, 2022 | Y | Y | N/A | Y | Y | Y | U | N/A | N/A | Y |

Y: Yes; N: No; U: Unclear; N/A: Not applicable

Q1: Is there congruity between the stated philosophical perspective and the research methodology?

Q2: Is there congruity between the research methodology and the research question or objectives?

Q3: Is there congruity between the research methodology and the methods used to collect data?

Q4: Is there congruity between the research methodology and the representation and analysis of data?

Q5: Is there congruity between the research methodology and the interpretation of results?

Q6: Is there a statement locating the researcher culturally or theoretically?

Q7: Is the influence of the researcher on the research, and vice-versa, addressed?

Q8: Are participants, and their voices, adequately represented?

Q9: Is the research ethical according to current criteria, or for recent studies, is there evidence of ethical approval by an appropriate body?

Q10: Do the conclusions drawn in the research report flow from the analysis or interpretation of the data?
